# Nonvirulent Infectious Salmon Anemia Virus (ISAV-HPR0) Not Detectable in Eggs or Progeny of Infected Captive Atlantic Salmon Brood

**DOI:** 10.3390/v16081288

**Published:** 2024-08-13

**Authors:** Mark P. Polinski, Demitri Lifgren, Richard D. Clayton, Janet V. Warg, Michael R. Pietrak, Brian C. Peterson

**Affiliations:** 1 National Cold Water Marine Aquaculture Center, U.S. Department of Agriculture–Agricultural Research Service, Franklin, ME 04634, USA; demitri.lifgren1@usda.gov (D.L.); michael.pietrak@usda.gov (M.R.P.); brian.peterson@usda.gov (B.C.P.); 2 National Veterinary Services Laboratories, U.S. Department of Agriculture–Animal and Plant Health Inspection Service, Ames, IA 50010, USA; richard.d.clayton@usda.gov (R.D.C.); janet.v.warg@usda.gov (J.V.W.)

**Keywords:** infectious salmon anemia virus (ISAV), hyper-variable region–0 (HPR0), vertical parent-to-offspring transmission, aquaculture

## Abstract

The potential for infectious salmon anemia virus (ISAV)—an internationally regulated pathogen of salmon—to transmit vertically from parent to offspring is currently unclear. While the highly virulent ISAV phenotype known as ISAV-HPRΔ has been observed intra-ova, evidence for vertical transmission of the avirulent ISAV phenotype known as ISAV-HPR0 is lacking. In this study, we identified ISAV-HPR0-infected Atlantic salmon broodstock during spawning within a government research recirculating aquaculture facility using qPCR. Eggs and milt from infected brood were used to initiate 16 unique family dam-sire crosses from which 29–60 fertilized eggs per cross were screened for ISAV using qPCR (limit of detection ~100 virus genome copies/egg). A portion of eggs (~300) from one family cross was hatched and further reared in biosecure containment and periodically screened for ISAV by gill clipping over a 2-year period. ISAV was not detected in any of the 781 eggs screened from 16 family crosses generated by infected brood, nor in 870 gill clips periodically sampled from the single-family cohort raised for 2 years in biocontainment. Based on these findings, we conclude that ISAV-HPR0 has a limited likelihood for vertical parent-to-offspring transmission in cultured Atlantic salmon.

## 1. Introduction

Infectious salmon anemia virus (ISAV) is an internationally regulated pathogen of salmon owing to its potential for high virulence and transmissibility. Although multiple species of salmonid are susceptible to ISAV, disease, known as infectious salmon anemia or ISA, is most notably observed in farmed Atlantic salmon (*Salmo salar*) of Norway, Canada, United Kingdom, Faroe Islands, and Chile [1]. Cumulative losses to production in these regions attributable to ISAV over the years can conservatively be estimated in the billions of USD (e.g., over USD 30 million in Canadian operations from one company in 2021–2022 alone [2]).

A complicating factor regarding both management and regulation of ISAV is the existence of two discrete virulence phenotypes. The disease-causing potential of ISAV appears to be driven primarily by the length of the hypervariable region (HPR) in Segment 6 of the viral genome that codes for the receptor-binding protein hemagglutinin-esterase [3]. Virus with a full-length HPR transcript (ISAV-HPR0) manifests as a nonvirulent phenotype that infects epithelia, typically within the gill and skin [4]. Virus with a shortened (deleted) HPR transcript (ISAV-HPR∆) has a truncated hemagglutinin-esterase stalk and altered receptor binding that produces a virulent phenotype that targets endothelial cells, particularly within membranes of the vascular system, and causes ISA [5]. While ISAV-HPR∆ can be readily cultured on multiple in vitro cell lines, ISAV-HPR0 has a nonsupportive invitrome making comparative study difficult [6].

The risk for nonvirulent (ISAV-HPR0) to virulent (ISAV-HPR∆) genetic/phenotypic mutation is an obvious area of concern for salmon farmers but is both temporally and mechanistically difficult to identify. There is a reasonably strong indication that ISAV-HPR0 can and does evolve into ISAV-HPR∆ in production environments [7]. However, some regions and production facilities with endemic HPR0 have remained free of HPR∆ for many years, indicating that mutation events might, at least in some instances, be rare [1]. The absence of an in vitro culture system for ISAV-HPR0 has hindered further experimental study into this issue. Without a model for further comparative study, it is difficult to accurately quantify risk. Nevertheless, current knowledge is sufficient to indicate that the risk is at least nonzero [3,8]. HPR0-free Atlantic salmon are, thus, preferred in virtually all cultured environments if presented as a no- or low-cost alternative for producers. 

One of the most straightforward strategies for reducing ISAV-HPR0 prevalence within Atlantic salmon production operations is ensuring discrete separation of cohorts with respect to virus transmission potential. To this end, an understanding of the parent-to-offspring vertical transmission of ISAV is critical. If intra-ova transmission is identified, then selecting offspring from virus-free broodstock is a straightforward strategy for ensuring virus-free progeny. If intra-ova transmission does not occur, eggs from unknown or infected broodstock could still be considered in establishing virus-free progeny, thereby reducing costs associated with testing and potential broodstock exclusion.

The potential for ISAV-HPR0 to be vertically transmitted is currently unclear. Both molecular and visual evidence for intra-ova infection of Atlantic salmon eggs has been shown for an ISAV-HPR∆ isolate (HPR-3) from Chile [9]. However, field evidence from the northeastern Atlantic during a 2007–2014 study suggests a general lack of ISAV-HPR0 vertical transmission from multiple European brood programs to progeny transferred to Faroe Island grow-out facilities [10]. In that study, ISAV-HPR0 sub-genotypes reported in egg-supplying broodfish from Iceland and Norway were of genetically discrete sub-genotypes compared to the ISAV-HPR0 detected in progeny fish that became infected in the Faroe Islands. Unfortunately, competitive advantage for the Faroese sub-genotype rather than lack of true vertical transmission is a possibility that confounds unequivocal determination for a lack of vertical transmission in this instance. This is potentially further confounded by a coinfection event detected in at least one of the sampled progeny populations in that study. 

In this study, our aims were to (i) ensure that current molecular detection assays for ISAV could be used to robustly identify virus within egg samples and (ii) identify with high confidence the likelihood for ISAV-HPR0 to be vertically transmitted from parent to offspring by screening fertilized eggs and progeny collected from confirmed ISAV-HPR0-infected Atlantic salmon broodstock.

## 2. Materials and Methods

Two discrete year-classes of Atlantic salmon broodfish housed at the U.S. Department of Agriculture–Agricultural Research Service’s National Cold Water Marine Aquaculture Center (NCWMAC) in Franklin, ME, were used in this study [11]. One population consisted of 4-year-old salmon that spawned in November of 2018. Eggs from these fish were reared through eye-up at NCWMAC, and on 11 February 2019, a portion of eggs from 6 dam-sire crosses were sent by courier to the U.S. Department of Agriculture’s Animal and Plant Health Inspection Service (APHIS) National Veterinary Services Laboratories (NVSL) in Ames, IA, for ISAV screening and rearing in ISAV-free biocontainment as described below. The second discrete population of fish consisted of 4-year-old salmon at NCWMAC that spawned in November of 2021. Eggs from these fish were reared at NCWMAC through eye-up, and on 28 January 2022, a portion of eggs from 10 dam-sire crosses were sampled and screened for ISAV at NCWMAC as described below. All animals held at NCWMAC were reared following Institutional Animal Care and Use Committee supervision and approval (ARS-NCWMAC Standard Operating Procedures: Care and Use of Research Animals, approved January 2018 and January 2021). All animals held at NVSL were reared following NVSL Institutional Animal Care and Use Committee supervision and approval (APH-2016-549 and APH-2019-821).

### 2.1. Identification of Infected Broodstock

In 2018, all NCWMAC broodfish were subject to ISAV screening of gill tissues following gamete collection. A sample of gill lamellae (~50 mg) was collected from the outermost right gill arch immediately after gamete stripping using sterile scissors and forceps. Samples were placed into 2 mL tubes containing 1 mL of RNAlater™ Stabilization Solution (ThermoFisher Scientific, Waltham, MA, USA). Ovarian fluid was also collected from stripped eggs using a 1 mL pipette, transferred to 1.5 mL tubes, and immediately frozen at −20 °C. Samples were shipped frozen to an ISAV Control Program APHIS-approved diagnostic laboratory (Kennebec River Biosciences (KRB), Richmond, ME, USA) for real-time reverse-transcriptase PCR (RT-PCR) screening and genotyping following the USDA-APHIS Maine Infectious Salmon Anemia Virus Control Program Standards [12].

In 2021, gill clips were similarly collected from all spawning adults following gamete collection, but with slight modifications relative to 2018. Specifically, gill samples were collected in duplicate and immediately frozen in liquid nitrogen followed by −80 °C storage. Ovarian samples were not assessed; however, duplicate samples of anterior kidney were collected immediately following fish euthanasia and frozen in liquid nitrogen as a proxy for identifying systemic infections. Duplicate samples of both kidney and gill were then screened for ISAV separately, where one replicate was sent to an APHIS-approved diagnostic laboratory (Kennebec River Biosciences) and the other replicate was screened in-house at the NCWMAC using the recently published multiplex real-time quantitative polymerase chain reaction (qPCR) methods for ISAV detection and phenotypic differentiation [13]. 

### 2.2. Spawning and Egg Incubation

In both 2018 and 2021, broodfish were palpated for ripeness 1–2 times per week during the spawning season (October–December) under tricaine mesylate (MS-222) anesthesia. Gametes were collected separately for males and females. Eggs were hand-stripped into 4 L plastic buckets. Milt was hand-stripped into 200 mL paper cups and immediately transferred to 200 mL tissue culture flasks, where 10 µL of sperm was visualized for motility by water activation. Approximately 237 mL (1 cup) of eggs per spawning female was transferred to a fine single-mesh strainer (Grainger) to drain ovarian fluid, then placed in a clean 4 L plastic bucket. Two mL of milt was added to fluid-free eggs followed immediately by a sperm activating solution (2.42 g Tris, 5.0 g NaCl, 3.75 g glycine in 1 L well-water; pH adjusted to 8.0) to a volume sufficient to cover the eggs. The eggs were mixed by swirling, allowed to sit for 5 min, and water-hardened for 1 h in ~1 L/min flowing fresh well-water. In 2021, spawning was conducted as for 2018 with the addition that fertilized eggs were subjected to an iodine-based disinfection procedure during water hardening as per standard industry practice consisting of a 50 µg/L Ovadine (Syndel, Ferndale, WA, USA) treatment for 30 min as per the manufacturer’s instructions. In 2018, no egg disinfection procedures were administered.

Once water-hardened, eggs were cultured in vertical tray incubators (MariSource, Burlington, WA, USA) with 8–11 °C recirculating well-sourced UV-treated fresh water. Family crosses were segregated into 4 per tray using mesh isolation baskets (MariSource). In 2018, eggs were incubated for approximately 450 degree-days before being shipped to NVSL for ISAV screening and rearing. In 2021, eggs were reared through eye-up (approximately 300 degree-days) before being sampled. 

### 2.3. Biosecure Progeny Rearing

Eggs and fish housed at NVSL were reared in a biosecure facility with a domestic drinking water source (carbon filtered and sodium thiosulfate treated to neutralize chlorine, and pH adjusted with 10% HCL to approximately pH 7.7). Eggs were incubated in vertical tray incubators identical to those used at NCWMAC upon arrival (15 February 2019). Flow-through water temperatures were 8–10 °C. Following hatching, all fish were reared in 123 L circular tanks from 21 March 2018, to 3 December 2019, during which time temperatures were increased from 9 to 14 °C. Fish were then divided equally into six 757 L circular tanks for the remainder of the trial, with water temperatures maintained between 10–15 °C. Fish in 3 of the tanks were smolted to 34 ppt saltwater using Instant Ocean Sea Salt (Instant Ocean Spectrum Brands, Blacksburg, VA, USA) over a 2-day period beginning 15 July 2020 (505 days post-hatch). 

### 2.4. ISAV Detection

ISAV screening of gill and ovarian fluid samples conducted at KRB and NVSL targeted genomic and messenger RNA from Segment 8 of the virus using quantitative PCR (qPCR) following the USDA-APHIS approved diagnostic protocol [14]. ISAV screening of gill samples and eggs conducted at NCWMAC were performed using a newly described multiplex qPCR assay [13]. Specifically, total RNA from ~20 mg gill samples and single whole eggs was extracted in TRI Reagent (ThermoFisher) as per manufacturer’s instructions, implementing a 5 mm steel bead and TissueLyser II (Qiagen, Hilden, Germany), operating for 2 min at 25 Hz. Specific to RNA extracted from eggs, an additional purification step was carried out on the eluted RNA to remove PCR inhibitors by adding one volume of 5 M ammonium acetate that was briefly vortexed, incubated at 4 °C for 30 min, and centrifuged at 16,000× *g* for 10 min. Supernatant was discarded, and pelleted RNA was re-eluted and used as template for qPCR assessments as previously described [13]. 

To ensure recovery efficiency as well as to determine a reliable limit of detection for ISAV-HPR0 in whole embryo samples, we utilized spike-in controls over a dynamic range of 7–7 × 10^4^ ISAV-HPR0 genome copies per sample. ISAV-HPR0 genetic material was obtained from a gill sample previously identified to be highly infected with ISAV-HPR0. Tissues were subjected to 3 freeze–thaw cycles, vortexed, and clarified by centrifugation at 10,000× *g* for 10 min at 4 °C. Supernatant was then used to create a 4-step 10-fold dilution series in PBS, and 10 µL of each dilution was spiked into triplicate embryo samples prior to RNA extraction and compared to extractions completed in the absence of embryos. 

### 2.5. Statistical Analysis

Prevalence was assessed based on sampling and testing F1 generation from crosses of broodstock testing positive for ISAV-HPR0 (purposive samples selected for vertical transmission potential) in two separate class cohorts [15,16] assuming 98% diagnostic test sensitivity based on diagnostic performance of similar qPCR assays [17,18,19].

## 3. Results

Two qPCR assays were effectively used to identify ISAV genetic material in both eggs and gill of Atlantic salmon in this study: the World Organisation for Animal Health (WOAH) reference assay employing primers and probe designed by Snow et al., 2006 [20], and a newer multiplex assay designed by Rounsville et al. [13], that differentially detects ISAV-HPR0 and ISAV-HPR∆. For 2021 broodstock gill samples where both assays were simultaneously performed, diagnostic agreement was 100%, with strong correlation in estimating relative quantitative loads (i.e., Ct values; Table S1, Supplementary Materials). The newer Rounsville et al. qPCR assay performed as expected, with a theoretical limit of detection of 1–3 copies per reaction, calculated from gblock-derived standards, similar to limits previously identified [13]. We further identified that our methods for extracting target viral RNA from individual whole eggs containing eyed embryos produced roughly equivalent extraction efficiencies for ISAV-HPR0 RNA (mean 124%; SD 54%) relative to nonegg samples (Table S2, Supplementary Materials). Thus, our theoretical experimental limit of detection was approximately 100–300 viral RNA genome copies per eyed-egg and approximately 10–30 copies per mg gill tissue. 

### 3.1. ISAV-HPR0 Detection in 2018 Broodfish and Resulting Embryos

ISAV-HPR0 RNA was detected in gills of six of the six dams and four of the five sires used from this cohort, which resulted in both parents being ISAV-HPR0-infected in five of the six crosses considered (Table S1; Supplementary Materials). The one instance where only the dam was ISAV-HPR0-infected was also the only instance where ISAV-HPR0 was detected in the ovarian fluid of the dam, indicating that, at least in this instance, eggs had been directly exposed to viral RNA. ISAV was not detected in any of the 181 embryos screened from these six family crosses. We assessed that this six-family embryo cohort was infected with ISAV-HPR0 at ≤2% prevalence with 95% confidence by the time of hatch (Table 1).

### 3.2. ISAV-HPR0 Detection in 2021 Broodfish and Resulting Embryos

ISAV-HPR0 was detected in gills of all 10 dams and 10 sires considered in this study within this year class (Table S1; Supplementary Materials). ISAV-HPR0 was detected in kidney samples indicative of systemic infections in six of the 10 sires and none of the 10 dams. ISAV-HPR0 was not detected in any of the 600 eyed embryos resulting from these 10 family crosses. We assessed that this embryo cohort was infected with ISAV-HPR0 at ≤1% prevalence with greater than 99% confidence at the time of sampling (Table 2).

### 3.3. ISAV-HPR0 Detection in 2019 Single Family Cohort Raised for 2 Years in Biocontainment

ISAV-HPR0 was not detected from any of the 20 alevins sampled four days post-hatch (dph), nor in any of the 870 gill clip samples collected over four sampling events at all subsequent life stages (Table 3). This included samples of fish held continuously in freshwater (n = 420) as well as for fish transitioned to saltwater (n = 420).

## 4. Discussion

We demonstrate in this study that both Snow et al.’s [20] and Rounsville et al.’s [13] qPCR assays could effectively be used to identify ISAV from eyed Atlantic salmon embryos. It should be noted that the extraction of total RNA using Tri Reagent from single whole embryos resulted in poor nucleic acid purity, as measured by spectrophotometric absorbance and evidence of qPCR-inhibition if subsequent ammonium acetate purification steps were omitted—an occurrence that we presume resulted from high quantities of protein within eggs, although this was not directly measured. Nevertheless, standard extraction methods with the addition of an ammonium acetate purification step demonstrated no loss of efficiency for obtaining target ISAV RNA relative to neat or in-tissue spike-ins and, thus, provides a highly sensitive method for identifying ISAV in salmon eggs.

Genomic material of ISAV-HPR0 was not detected in any of the 781 live embryos screened in this study. Although it could be argued that individual eggs might have been infected at a concentration below the limit of detection of this assay (~100 virus genome copies per egg), the stochastic probability for single-copy inclusion in the 781 tested subsample coupled with the high analytical sensitivity of the assay suggests that if low-load transmission was occurring, it was extremely rare. Indeed, the probability for vertical transmission to have occurred within the tested embryo population can conservatively be calculated at less than 0.6% with greater than 99% confidence in this study. A low likelihood for low-load vertical transmission is further supported by a complete lack of detection over a 2-year period in repeated sampling of the single-family cohort obtained from an ISAV-HPR0-infected dam. The lack of detection in this single-family cohort (and the 2018 cohort in general) is particularly significant as no egg-disinfection protocols were implemented despite the presence of ISAV-HPR0 genetic material being identified in the ovarian fluid of the dam. Environmental stability of ISAV is low (e.g., hours) [21], as is typical of enveloped orthomyxoviruses [22]. This would suggest that virus material was restricted to the extra-ova matrix and, thus, it is probable that even in the presence of infected ovarian fluid, the mother-to-offspring transmission potential of ISAV-HPR0 is low and can be further mitigated by standard egg disinfection protocols. 

Previous investigation of one Faroese and two Icelandic commercial Atlantic salmon broodstock facilities over an 8-year period (2007–2014) indicated a lack of ISAV-HPR0 vertical transmission as evidenced by positive detections within progeny (when subsequently identified) of a different genetic subgroup relative to isolates sequenced from infected parental brood [10]. The authors concluded that ISAV-HPR0 vertical transmission was uncommon—a conclusion supported by this study. Indeed, data from our study indicate that “uncommon” may be interpreted to be <1% probability in an aquaculture setting employing standard commercial methods with or without egg disinfection protocols.

## Figures and Tables

**Table 1:** 2018 broodfish and embryo ISAV-HPR0 detection. A qPCR threshold cycle (Ct) for ISAV-HPR0 detection in gill and ovarian fluid samples of broodfish is presented for each mating pair (embryo set). qPCR screening outcome of eyed embryos is also presented, as is within-set and within-cohort 95% confidence probabilities for ISAV-HPR0 prevalence. OvF = ovarian fluid; U = undetermined; ND = not detected.

Embryo Set	Dam (Gill Ct, OvF Ct)	Sire (Gill Ct)	Embryos Screened	Screening Outcome	Infection Prevalence (95% Confidence)	Cohort Infection Prevalence (95% Confidence)
1	1 (27.5, U)	1 (30.0)	29	ND	<10%	
2	2 (27.9, U)	1 (30.0)	31	ND	<10%	
3	3 (27.0, U)	2 (33.1)	30	ND	<10%	<2%
4	4 (29.0, U)	3 (32.1)	30	ND	<10%	
5	5 (29.9, U)	4 (28.4)	31	ND	<10%	
6	6 (29.4, 33.5)	5 (U)	30	ND	<10%	

**Table 2:** 2021 broodfish and embryo ISAV-HPR0 detection. A qPCR threshold cycle (Ct) for ISAV-HPR0 detection in gill and kidney samples of broodfish is presented for each mating pair (embryo set). qPCR screening outcome of embryos is also presented, as is within-set 95% and cumulative within-cohort 99% confidence probabilities for ISAV-HPR0 prevalence. Kid. = kidney; U = undetermined; ND = not detected.

Embryo Set	Dam (Gill Ct, Kid. Ct)	Sire (Gill Ct, Kid. Ct)	Embryos Screened	Screening Outcome	Infection Prevalence (95% Confidence)	Cohort Infection Prevalence (99% Confidence)
7	7 (28.4, U)	6 (19.2, 35.9)	60	ND	<5%	
8	8 (31.8, U)	7 (14.4, 35.3)	60	ND	<5%	
9	9 (28.8, U)	8 (29.3, 37.7)	60	ND	<5%	
10	10 (23.9, U)	9 (33.7, U)	60	ND	<5%	
11	11 (28.5, U)	10 (19.4, 36.8)	60	ND	<5%	<1%
12	12 (36.1, U)	11 (33.9, U)	60	ND	<5%	
13	13 (25.7, U)	12 (26.0, 34.7)	60	ND	<5%	
14	14 (28.0, U)	13 (28.0, 36.0)	60	ND	<5%	
15	15 (28.4, U)	14 (26.5, U)	60	ND	<5%	
16	16 (27.4, U)	15 (30.9, U)	60	ND	<5%	

**Table 3:** Single-family cohort ISAV-HPR0 detection post-hatch. ISAV-HPR0 qPCR detection by lethally sampling a portion of whole fish sans eyes/yolk sac (4 dph) or nonlethally by gill clip (all other timepoints). Within-sample group and cumulative within-cohort 95% confidence probabilities for ISAV-HPR0 prevalence is indicated. ND = not detected.

Days Post-hatch	Life Stage	Sample Type	Samples Screened	Screening Outcome	Infection Prevalence (95% Confidence)	Cohort Prevalence (99% Confidence)
4	Alevins	Whole fish ^1^	20	ND	<16%	
99	Parr	Gill	30	ND	<11%	
506	Smolt/juvenile	Gill	280	ND	<1%	<1%
596	Smolt/juvenile	Gill	280	ND	<1%	
685	Adult	Gill	280	ND	<1%	

^1^ Yolk sac and eyes removed.

## Data Availability

All data generated and described as part of this manuscript are provided in Supplementary Materials.

## Supplementary Materials

The following supporting information can be downloaded at: https://www.mdpi.com/article/10.3390/v16081288/s1, Table S1: Broodstock testing; Table S2: Efficiency testing; Table S3: Embryo testing; Table S4: Progeny testing.
